# Individual mandibular movement registration and reproduction using an optoeletronic jaw movement analyzer and a dedicated robot: a dental technique

**DOI:** 10.1186/s12903-020-01257-6

**Published:** 2020-10-07

**Authors:** Massimo Carossa, Davide Cavagnetto, Paola Ceruti, Federico Mussano, Stefano Carossa

**Affiliations:** CIR Dental School, Department of Surgical Sciences, University of Turin, Turin, Italy

## Abstract

**Background:**

Fully adjustable articulators and pantographs record and reproduce individual mandibular movements. Although these instruments are accurate, they are operator-dependant and time-consuming. Pantographic recording is affected by inter and intra operator variability in the individuation of clinical reference points and afterwards in reading pantographic recording themselves. Finally only border movements can be reproduced.

**Methods:**

Bionic Jaw Motion system is based on two components: a jaw movement analyzer and a robotic device that accurately reproduces recorded movements. The jaw movement analyzer uses an optoelectronic motion system technology made of a high frequency filming camera that acquires 140frames per second and a custom designed software that recognizes and determines the relative distance at each point in time of markers with known geometries connected to each jaw. Circumferential modified retainers connect markers and do not cover any occlusal surfaces neither obstruct occlusion. The recording process takes 5 to 10 s. Mandibular movement performance requires six degrees of freedom of movement, 3 rotations and 3 translations. Other robots are based on the so-called delta mechanics that use several parallel effectors to perform desired movements in order to decompose a complex trajectory into multiple more simple linear movements. However, each parallel effector introduces mechanical inter-component tolerances and mathematical transformations that are required to transform a recorded movement into the combination of movements to be performed by each effector. Bionic Jaw Motion Robot works differently, owing to three motors that perform translational movements and three other motors that perform rotations as a gyroscope. This configuration requires less mechanical components thus reducing mechanical tolerances and production costs. Both the jaw movement analyzer and the robot quantify the movement of the mandible as a rigid body with six degrees of freedom. This represents an additional advantage as no mathematical transformation is needed for the robot to reproduce recorded movements.

**Results:**

Based on the described procedure, Bionic Jaw Motion provide accurate recording and reproduction of maxillomandibular relation in static and dynamic conditions.

**Conclusion:**

This robotic system represents an important advancement compared to available analogical and digital alternatives both in clinical and research contexts for cost reduction, precision and time saving opportunities.

## Background

The need of perfecting the registration and transfer of jaw relations starts with the development of complete removable dentures. The first system that allowed to evaluate stone models statically at a given vertical dimension of occlusion (VDO) was described by Gariot in 1805 [1, 2]. Since then, a constant progression led to the development of modern dental articulators and facebows. Daniel T Evens (1840) introduced protrusive and lateral movements, while Bonwill (1858), a mathematician, built the first mean value articulator. William Earnest Walker (1856), developed the “clinometer”, the first example of kinematic facebow to reproduce condylar inclination, and the first semi adjustable articulator. Gysi-Muller (1896–1899) constructed an articulator mimicking the form of the condyle and glenoid fossa [3, 4]. During the first half of the XX century articulators had a rapid development (Table 1) reproducing more and more accurately the individual border movements. During the Sixties the first fully adjustable articulators and pantograph facebows appeared, among which the most used and known systems were Hanau 130–21 [5, 6], Stuart’ s articulator [7] that was called the gnathological computer and Denar D5A [3, 8]. They presented components that could be adjusted to reproduce individual condylar movements as a main innovation compared to semi adjustable that presented standardized flat tracks and planes [9]. Unfortunately, fully adjustable articulators require more complex records (i.e. pantographic and stereographic tracings) and therefore need more time to be programmed [10]. Notwithstanding their precision, these devices are hindered by several limitations. The first possible source of error is the ability of the clinician to measure articulator settings from the pantographic tracings [11]. Other limits are linked to the difficulty of the mechanical components to reproduce movements generated by complex three dimensional structures like the ones of the condyle and the glenoid fossa [12, 13]. Other issues possibly preventing the optimal reproduction of border movements could be:

- the identification of the correct location of the reference plane angle [14],
- the assumption that at least in the first millimeters the mandible makes a pure rotation around its hinge axis [15],
- the interoperator and intraoperator variability of measurements [11].

**Table 1:** Table summarising the main examples of articulators and their evolution through time

**Huberty** articulator	1901	**Hanau** model H110 articulator	1926
**Kerr** articlator	1902	**Philips** student articulator (Model C)	1926
**Christensen**'s articulator	1905	**Hanau** model H110 modified articulator	1927
New century **George Snow**	1906-1907	**House** articulator	1927
**The Acme** articulator	1906	The **Stansberry** tripod instrument	1929
**Gysi adaptable** articulator	1910	**Gysi Truebyte** articulator	1930
**Luce** articulator	1911	**Terrell**'s precision co-ordinator	1930
**Eltner** articulator	1912	**Hanau** crown and bridge articulator	1934
**Gysi simplex** articulator	1914	The **Phillips** occlusoscope	1938
**Alligator-Rubert Hall**	1915	The **McCollum** gnathoscope	1939
**Hall**'s anatomic articulator	1915	**Stephan** articulator modified	1940
**Gysi Dreipunkt** articulator	1917	**Stephan** articulator model P	1940
**Monson**-maxillomandibular instrument	1918	The **Fournet** articulator	1940
**Hagman** balancer	1920	**Dentatus** articulator ARH model	1944
**Stephan** articulator	1921	**Johnson-Oglesby** articulator	1950
**Hanau** articulator	1921	**Moyer** articulator	1950
**Hanau** model M **kinoscope**	1923	**Coble** articulator	1950
The **Homer** relater	1923	**Bergstorm** articulator	1950
**Wadsworth** articulator	1924	**The Galetti articulator**	1950-1960

A substantial improvement regarding intra and inter operator agreement of recorded values was achieved with the introduction of the digital pantograph Denar Cadiax Compact (Teledyne Waterpik) [16] and Arcus Digma (KaVo America) [17] that is a jaw motion analyzer that uses multiple ultrasonic to record mandibular movements. Besides, movements that can be recorded and reproduced are stereotyped and they do not reflect dynamicity of functional movements. Since the 1990s there has been growing interest to overcome the above mentioned limitations with jaw robots [18, 19]. To the authors’ knowledge, there are only two systems in dental literature that tried to register and reproduce individual mandibular movement for clinical purposes but are limited to digital simulation of individual mandibular movements within a virtual environment [20]. The first one uses a CAD software called Adams to analyze data about mandible position that is obtained using an optoelectronic motion capturing system (370 frames per second) that records the light reflected from six point of reference whose position in relation to the mandible is known. The proposed method follows a geometrical study of the subject’s mandibular and maxillary teeth. It records chewing paths using an optoelectronic motion-tracking technology [21]. These devices were originally developed to record tongue and mouth movements for speech research [21, 22]. The second one uses a facial scanner target tracking. Eight targets are positioned on both maxillary and mandibular incisors to record mandibular movements. Mandibular movements are reconstructed after having eliminated head parasite movements that are the ones recorded from the maxilla. A computer software (Exocad, GmbH) allows to evaluate occlusal contacts.

## Methods

**Fig. 1:**
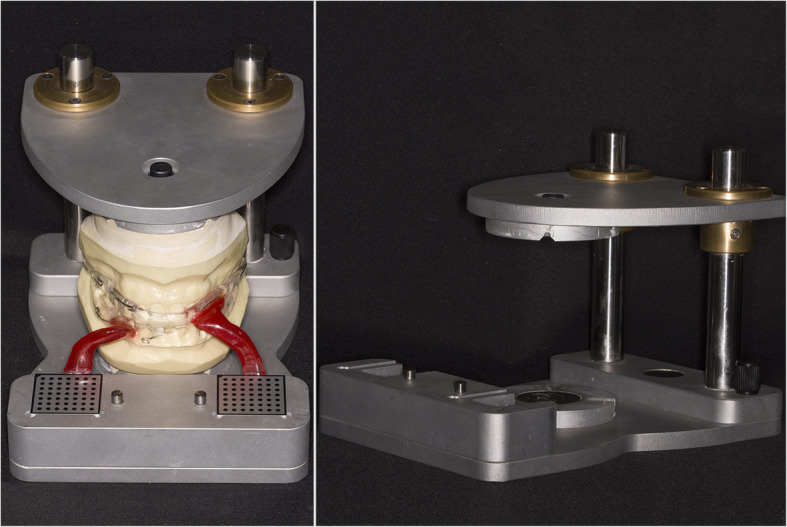
Installation aid. The Installation aid is a device meant to position the markers in a known position in relation to each jaw: on the right side the installation aid without the casts, on the left side the casts mounted on the installation aid with maxillary and mandibular retainers

**Fig. 2:**
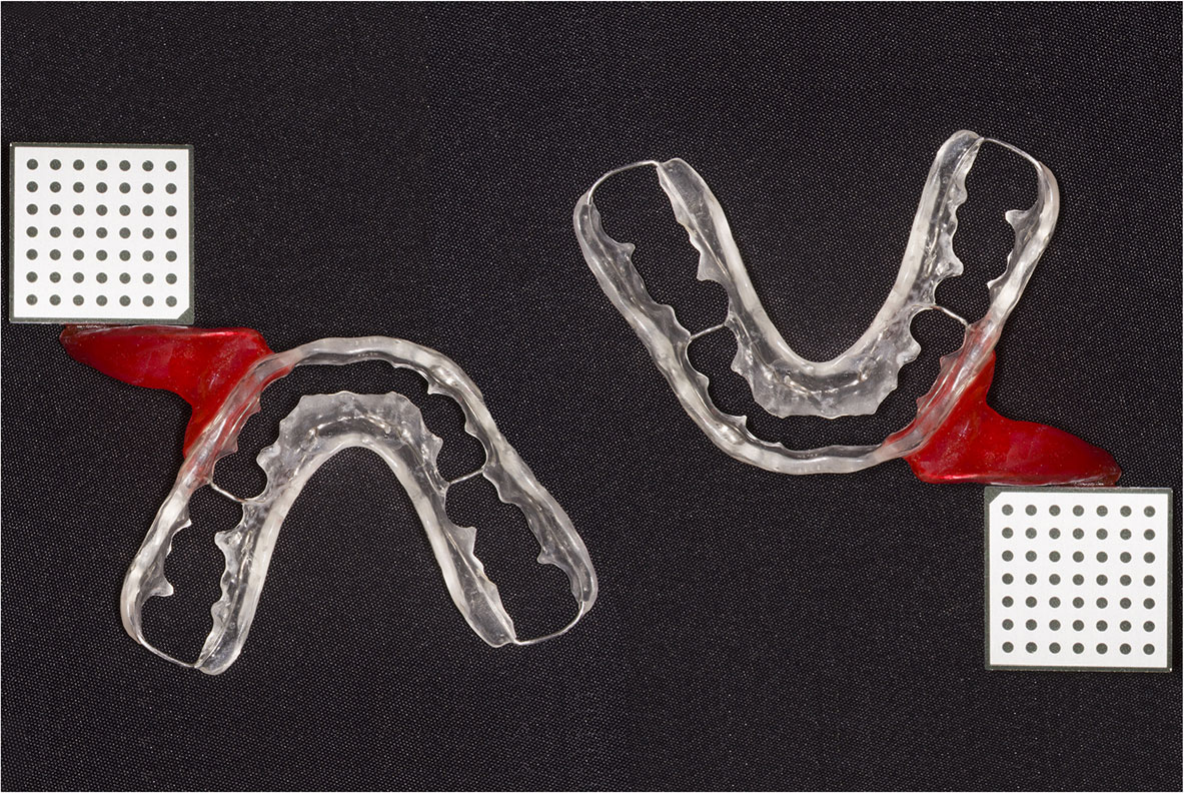
Maxillary and mandibular retainers. They need to have occlusal clearance. This design is suitable in absence of severe deep-bite. The resin vestibular to the 5th sextant and lingual to the 2nd sextant is removed in patients with severe deep-bite to allow maximum intercuspation and eccentric movements with no interference

**Fig. 3:**
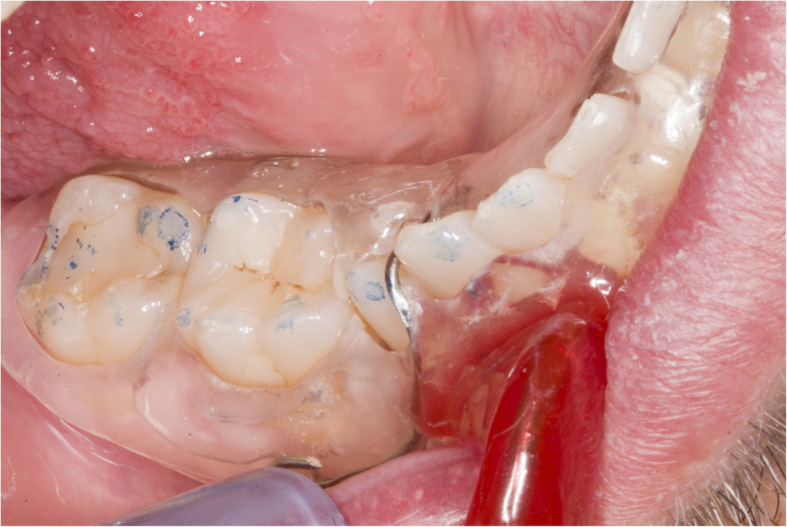
Intraoral check of the retainers. It is necessary to check there are no occlusal contacts on the retainers during function. The picture depicts the contact points of the patient in the 4th quadrant. No contact points should be present on the splint surface

**Fig. 4:**
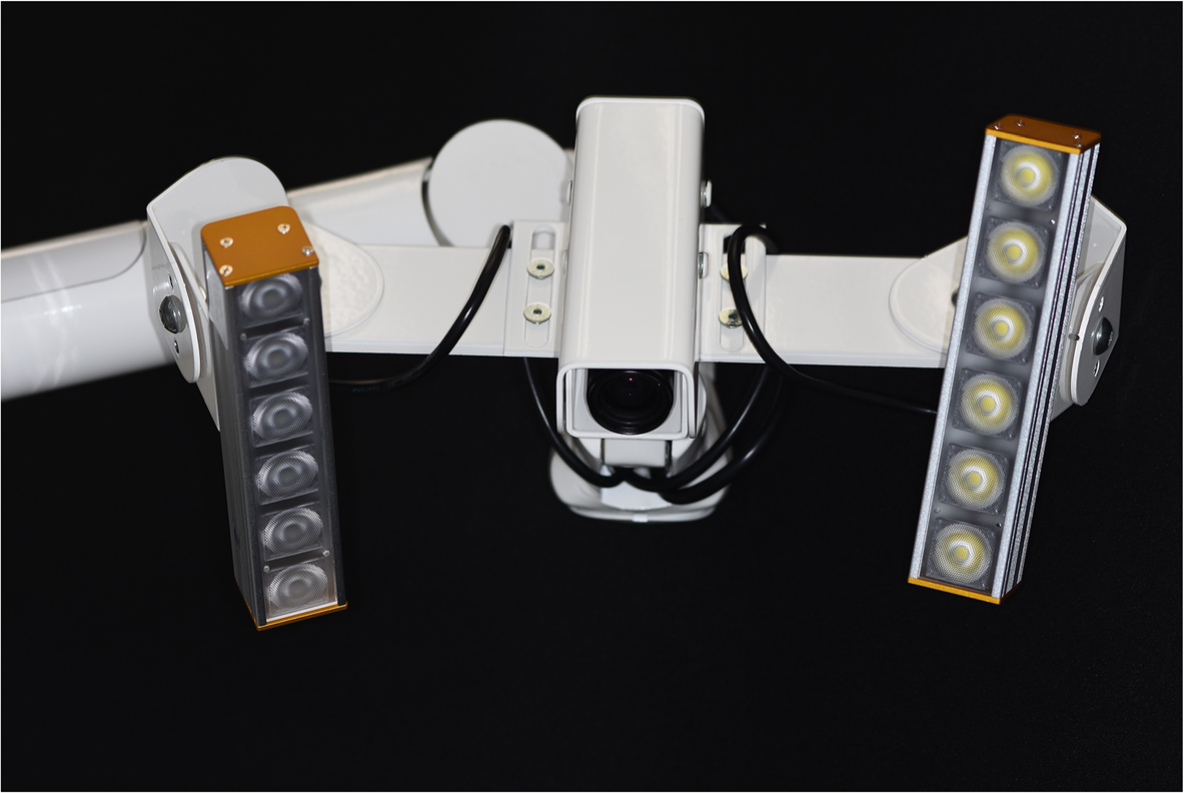
Bionic Jaw Motion Movement Analyzer. The jaw movement analyzer is composed of a high-speed recording camera and a software running on a computer. The software recognizes the known geometries of the markers and their optical deformation during movement registration thus reconstructing mandibular movement. It is mandatory to check whether the artificial vision software is able to locate reciprocal position of the markers during all opening phases before performing the actual recording (see fail number in Video 1, to ensure the best possible recording no fail should occur)

**Fig. 5:**
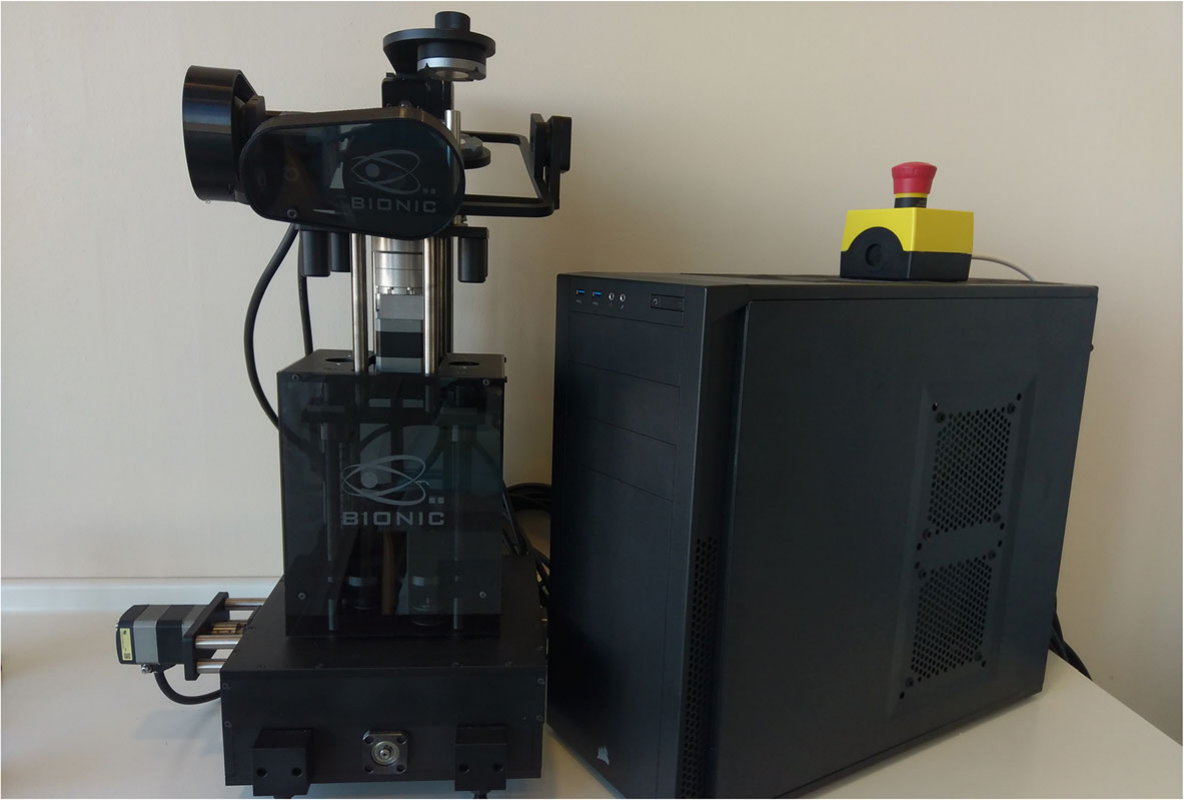
Bionic Jaw Motion robot. The robot with its computer unit that controls the motors and makes the robot move

**Fig. 6:**
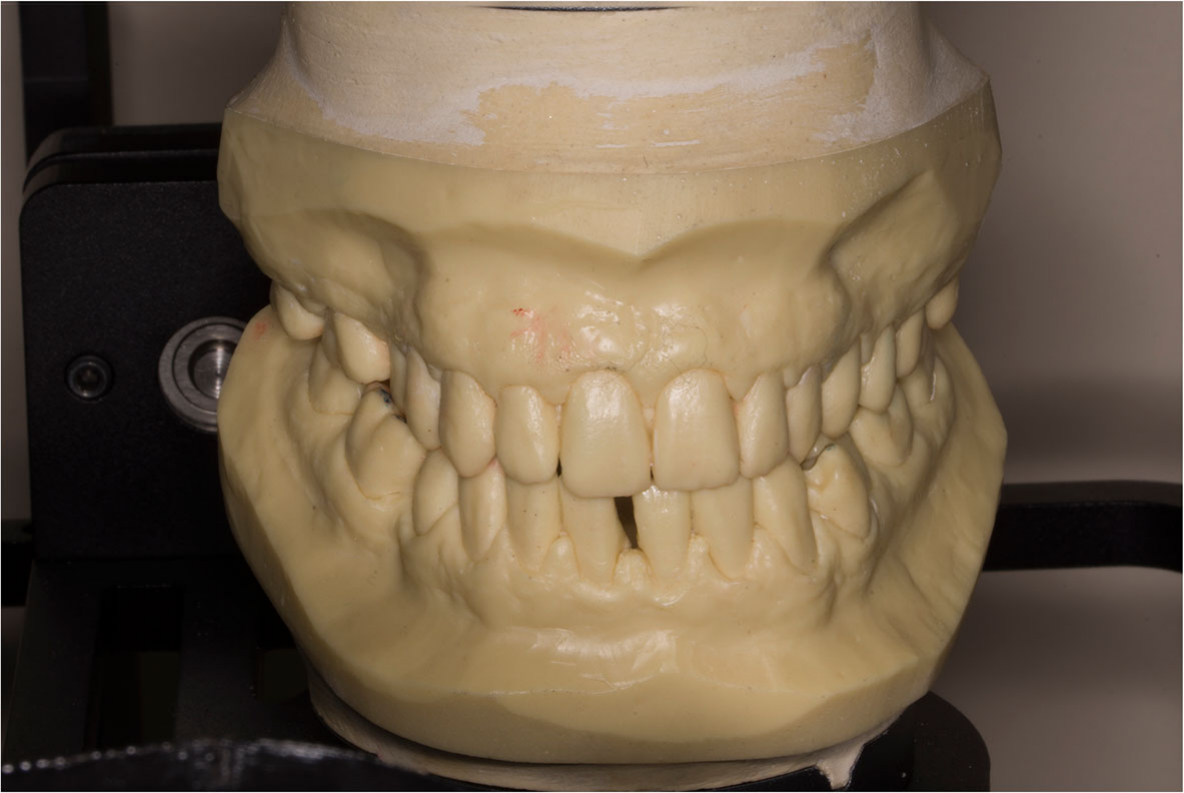
Models set in the cast holders. Close up image of the upper and lower resin models in the robot cast holders

**Fig. 7:**
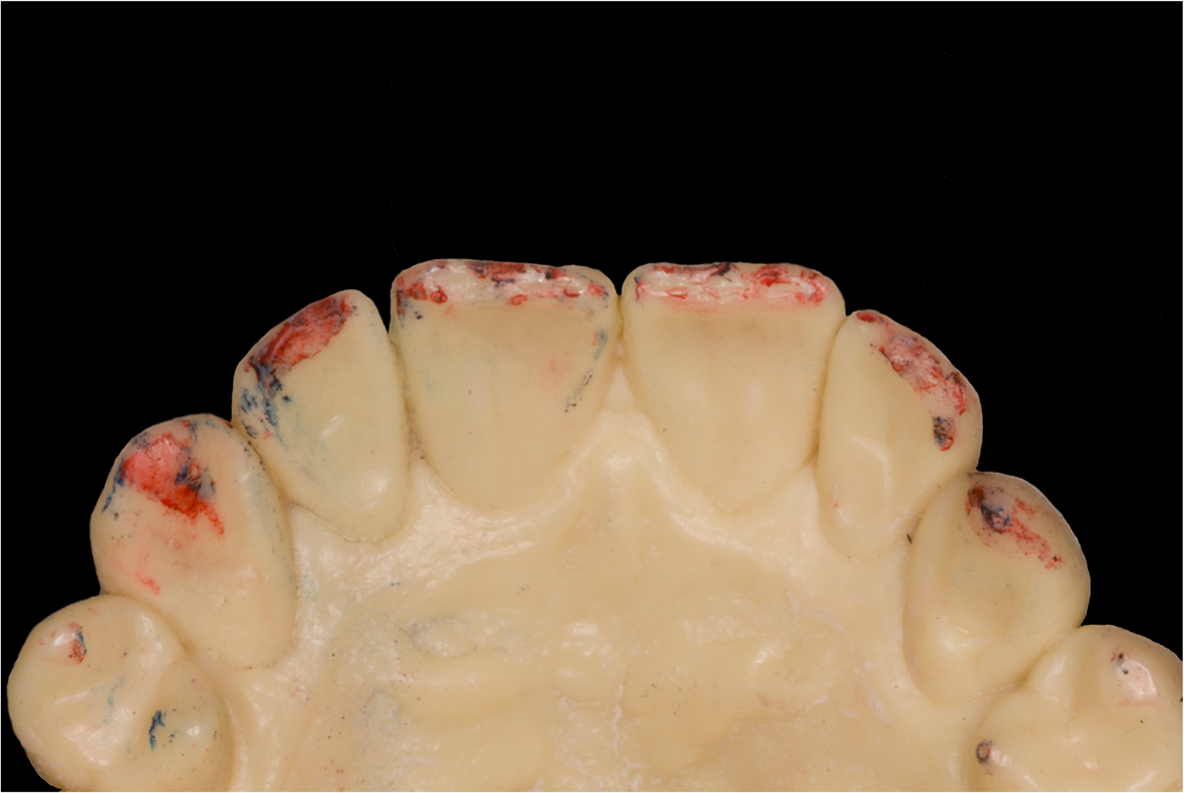
Example of occlusal contact during laterotrusion. Contact points during right laterotrusion on the models moved with the robot

The presented system is called Bionic Jaw Motion (BMJ; Bionic Technology, Vercelli, Italy) and it is composed of a Jaw movement analyzer and a robotic articulator. Since our study design is a report of a dental technique, no ethical approval was gained in accordance to EU regulations [23, 24]. The volunteer whose reports were included in this study signed a written informed consent to undergo the examination and to eventually make his examination available for research purposes. The acquisition system is similar to the aforementioned ones. It uses a technology, based on high frames-per-second filming, that through an artificial vision system is capable to achieve higher precision because it is capable of computing a high amount of information. In particular, it can dimension and quantify the spatial position of known geometries applied to markers. The recording process of each acquisition last from 5 to 10 s depending on clinical requirements (Video 1). More than one acquisition can be performed but is not always required. Square markers with peculiar geometries on them are placed at a known position (Figs. 1 and 2) from each other and from maxillary and mandibular teeth, to which they are connected using a designed jig through respectively a maxillary and a mandibular circumferential retainer that do not interfere with occlusion and function (Fig. 3). The artificial vision system (Fig. 4) is capable of recognizing the geometric landmarks of the markers was developed by the automotive industry to plot the planarity of car components and adapted for dental purposes. Despite modern high-speed cameras can reach 2000 fps, the acquisition system is set to 140 fps to quantify movement. This choice was made after empirical laboratory data and previously published data on mandibular velocity [25]. Highest mandibular velocity in opening/closing phases ranges between 10 and 13 cm/s approximately [25, 26]. The system’s dimensions have been designed to guarantee precision with an accuracy to less than a tenth of a millimeter. The markers known position allows it to reach high precision during movement registration. A software elaborates data from markers position and digitalizes movements, that can be visualized as kinesiographic tracings and as three-dimensional relationship between virtualized models during recorded movements (Video 2). Current available instrumentation has limitations regarding its capability of reproducing complex trajectories determined by irregular geometries of the condyles and glenoid fossae and coherence of reference system between the patient and the mechanical instrument [27]. To the authors’ knowledge, BJM is the first system to have an integrated software designed to reproduce the recorded functional movements on a robotic jaw simulator (Figs. 5 and 6). In order to reproduce anatomical movements accurately, robots ought to have 6 degrees of freedom of movement, 3 translations and 3 rotations, with high movement accuracy [28]. The first prototypes of robots for clinical purposes were built using delta mechanics (Video 3), also called parallel robots [29]. Among their favorable characteristics one may enumerate the limited volume and fast operation modalities. On the other hand, their mechanics are complex. The numerous connections between each component demand a production system enabling particularly low mechanical tolerances, which is very expensive. To overcome delta mechanics limitations, BJM uses a different mechanical configuration. Complex effectuators are substituted by a simplified system comprised of three motors that work in translation and three motors that work in rotation as a gyroscope converging on the rotor that is the lower model holder. All rotations and all translations converge on the lower model holder thus conferring to it six degrees of freedom of movement (Video 3) [30]. Since even the acquisition system quantifies movement homogeneously through relative position of maxillary and mandibular markers, no mathematical transformation is needed to move the robot. This does not happen in robots designed with delta mechanics because mathematical transformations are required to break down movement in every effector axis [29]. These characteristics allow to obtain an excellent precision (Figs. 7 and 8 Video 4) and to contain prices for robot production.

**Fig. 8:**
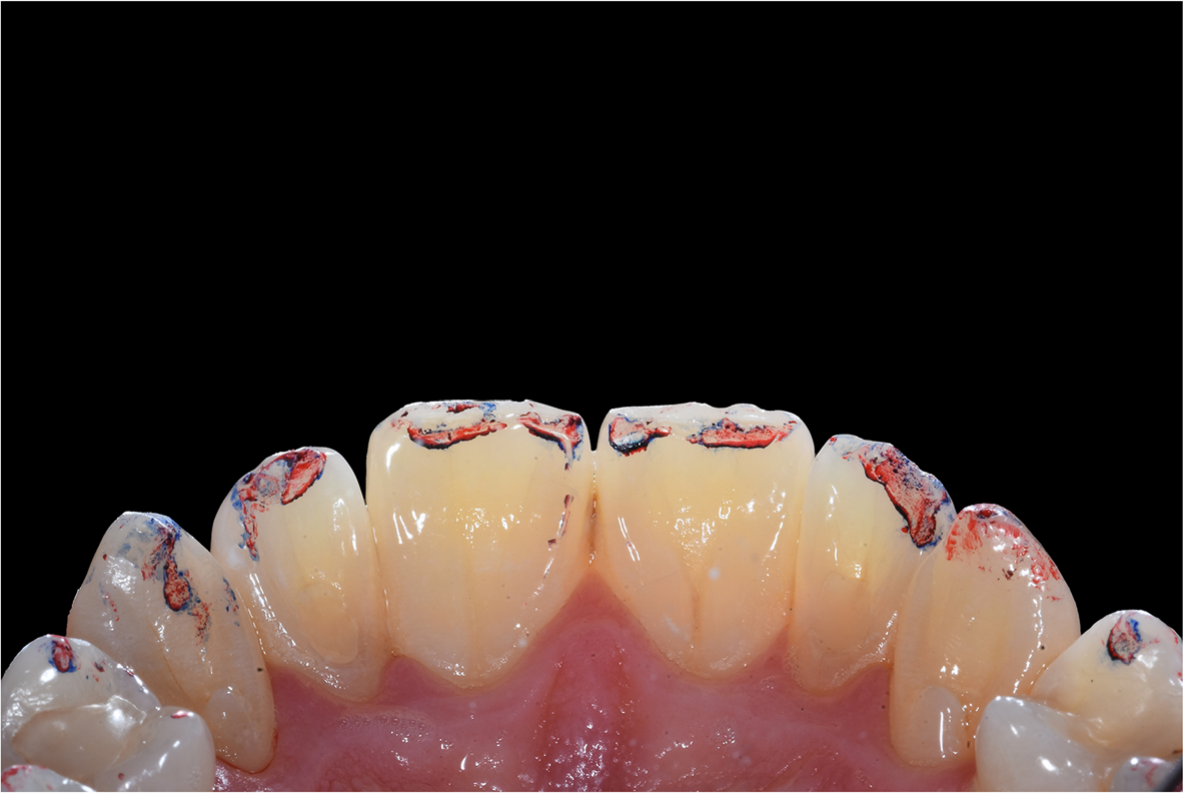
Intraoral check of contact points during the same movement. Contact points during right laterotrusion in patient’s mouth

## Discussion

BJM differs from other digital robotic systems for acquisition and reproduction of mandibular movement because it is the first, at least to Authors’ knowledge, that is capable to quickly record individual functional movement, analyze data, and reproduce it on a robot. Most published prototypes focus only or on movement recording or on robotic movement reproduction, usually using data arbitrarily inserted in a software and trying to reproduce them at best on a robot [28]. The technology of BJM allows to reproduce mandibular kinematics without being limited to stereotyped movements. It uses intraoral landmarks to quantify movement not being influenced by an external reference system (hinge axis) such as facebow- articulator systems, or by alteration of motion of the condyles, i.e. Articular Disc Displacement. It has an optimal intra- and inter-operator repeatability and reproducibility as the human factor is reduced as low as reasonably achievable. Another advantage is the shorter chair time and consequently lower cost for individual registration compared to pantographic tracings and articulator setting (few seconds vs several minutes or hours). Compared to other systems like Arcus Digma, BJM is considerably lighter and comfortable for the patient. It could represent a novel valuable tool for prosthetic, gnathological and orthodontic application both for clinical and for research purposes. For instance, this new method could provide easy and quick jaw movement recording in patients that need to undergo prosthetic rehabilitation and accurate jaw movements reproduction during laboratory phases. It could also prove itself useful in the study and diagnosis of tempo-mandibular disorders. It can be helpful in studying mandibular kinematics during speech and during other functional activities that are of interest, for example, as orthodontic research topic to study the relation between different jaw movement patterns and the development of alterations in maxillofacial growth.

## Conclusion

BJM quickly records and reproduces individual mandibular movements and overcomes many of the limitations of traditional pantograph-individual articulators systems. An intraoral reference system is adopted to avoid any possible mistake in clinical identification of extraoral landmarks whose univocal determination is nearly impossible. BJM also allows the recording of functional movement besides border movements.

## Supplementary information

**Additional file 1**

**Additional file 2**

**Additional file 3**

**Additional file 4**

## Data Availability

All data and materials are available from the corresponding author upon reasonable request.

## Abbreviations

BJM: Bionic Jaw Motion
VDO: Vertical dimension of occlusion
fps: Frames per second

## References

[1] Starcke EN (2006). The history of articulators: a perspective on the early years. Part I J Prosthodont.

[2] Starcke EN (2012). The history of articulators: a perspective on the early years, part 2. J Prosthodont.

[3] Starcke EN, Engelmeier RL (2016). The history of articulators: the wonderful world of “grinders”, Part III. J Prosthodont.

[4] Engelmeier RL, Belles DM, Starcke EN (2017). The history of articulators: the contributions of Rudolph L. Hanau and his company-part II. J Prosthodont.

[5] Javid NS, Porter MR (1975). The importance of the Hanau formula in construction of complete dentures. J Prosthet Dent.

[6] Tregaskes JN (1982). The procedures involved in the use of the Hanau 130–21 articulator.

[7] Stuart CE (1979). Use of the Stuart articulator in obtaining optimal occlusion. Dent Clin N Am.

[8] Ebel HE, Guyer SE, Lefkowitz W (1976). Reliability of fully adjustable, articulators using a computerized analysis. J Prosthet Dent.

[9] Bellanti ND (1973). The significance of articulator capabilities. I. Adjustable vs. semiadjustable articulators. J Prosthet Dent.

[10] Myers GE (1974). Status report on articulators. J Am Dent Assoc.

[11] Curtis DA, Sorensen JA (1986). Errors incurred in programming a fully adjustable articulator with a pantograph. J Prosthet Dent.

[12] Tryde G, McMillan DR, Christensen J, Brill N (1976). The fallacy of facial measurements of occlusal height in edentulous subjects. J Oral Rehabil.

[13] Christensen LV, Slabbert JC (1978). The concept of the sagittal condylar guidance: biological fact or fallacy?. J Oral Rehabil.

[14] Price RB, Gerrow JD, Ramier WC (1989). Potential errors when using a computerized pantograph. J Prosthet Dent.

[15] Ferrario VF, Sforza C, Miani A, Serrao G, Tartaglia G (1996). Open-close movements in the human temporomandibular joint: does a pure rotation around the intercondylar hinge axis exist?. J Oral Rehabil.

[16] Pelletier LB, Campbell SD (1991). Comparison of condylar control settings using three methods: a bench study. J Prosthet Dent.

[17] Park C (2016). Application of ARCUS digma I, II systems for full mouth reconstruction: a case report. J Dent Rehabil Appl Sci.

[18] Takamori T, Tsuchiya K (1993). Robotics, mechatronics and manufacturing systems.

[19] Xu W, Bronlund JE (2010). Mastication robots: biological inspiration to implementation.

[20] Kim J-E, Park J-H, Moon H-S, Shim J-S (2019). Complete assessment of occlusal dynamics and establishment of a digital workflow by using target tracking with a three-dimensional facial scanner. J Prosthodont Res.

[21] Röhrle O, Waddell JN, Foster KD, Saini H, Pullan AJ (2009). Using a motion-capture system to record dynamic articulation for application in CAD/CAM software. J Prosthodont.

[22] Guiard-Marigny T, Ostry DJ (1997). A system for three-dimensional visualization of human jaw motion in speech. J Speech Lang Hear Res.

[23] Directive 2001/20/EC of the European Parliament and of the Council of 4 April 2001 on the approximation of the laws, regulations and administrative provisions of the member states relating to the implementation of good clinical practice in the conduct of clinical trials on medicinal products for human use. Med Etika Bioet. 2002;9(1–2):12–9. 16276663

[24] European Commission - European Medicines Agency. Report on the conference on the Operation of the Clinical Trials Directive (Directive 2001/ 20/EC) and Perspectives for the Future, Conference held on 3 October 2007 at the EMEA, London (Report issued on November 30, 2007; Doc. ref.: EMEA/565466/2007). Available online: https://ec.europa.eu/health/sites/health/files/files/pharmacos/docs/doc2007/2007_11/ec_emea_conference_on_clinical%2520_trials_en.pdf.

[25] Karlsson S, Carlsson GE (1990). Characteristics of mandibular masticatory movement in young and elderly dentate subjects. J Dent Res.

[26] Karlsson S, Persson M, Carlsson GE (1991). Mandibular movement and velocity in relation to state of dentition and age. J Oral Rehabil.

[27] Brown T (1965). Physiology of the mandibular articulation. Aust Dent J.

[28] Bando E, Nishigawa K, Nakano M, Takeuchi H, Shigemoto S, Okura K, Satsuma T, Yamamoto T (2009). Current status of researches on jaw movement and occlusion for clinical application. Jap Dent Sci Rev.

[29] Schäfer P, Schiehlen W (1993). Application of parallel computing to robot dynamics. Robot Mechatronics Manuf Syst.

[30] Brown D, Peck M. Energetics of control moment gyroscopes as joint actuators. J Guid Control Dyn. 2009. 10.2514/1.42313.

